# Effect of *CX3CL1/CX3CR1* gene polymorphisms on the clinical efficacy of carboplatin therapy in Han patients with ovarian cancer

**DOI:** 10.3389/fgene.2022.1065213

**Published:** 2023-01-06

**Authors:** Xin-Chen Wang, Hong Zhou, Wen-Jing Jiang, Peng Jiang, Yan-Cai Sun, Wei-Jian Ni

**Affiliations:** ^1^ Department of Pharmacy, Anhui Provincial Cancer Hospital, The First Affiliated Hospital of USTC, Division of Life Sciences and Medicine, University of Science and Technology of China, Hefei, Anhui, China; ^2^ Department of Gynecological Oncology, Anhui Provincial Cancer Hospital, The First Affiliated Hospital of USTC, Division of Life Sciences and Medicine, University of Science and Technology of China, Hefei, Anhui, China; ^3^ Department of Pharmacy, Anhui Provincial Hospital, The First Affiliated Hospital of USTC, Division of Life Sciences and Medicine, University of Science and Technology of China, Hefei, Anhui, China; ^4^ Inflammation and Immune Mediated Diseases Laboratory of Anhui Province, the Key Laboratory of Anti-inflammatory of Immune Medicines, Ministry of Education, Anhui Institute of Innovative Drugs, School of Pharmacy, Anhui Medical University, Hefei, Anhui, China

**Keywords:** ovarian cancer, CX3CL1, CX3CR1, gene polymorphism, carboplatin

## Abstract

Gene polymorphisms have a close relationship with the clinical effects of carboplatin for ovarian cancer. Here, we investigated the relationship between *CX3CL1* and *CX3CR1* genotypes and the clinical efficacy of carboplatin in ovarian cancer, thereby clarifying the unidentified genetic factors that influence the efficacy of carboplatin in ovarian cancer. Based on the above purposes, we used Sequenom Mass ARRAY technology to detect *CX3CL1* and *CX3CR1* gene polymorphisms in 127 patients with carboplatin-treated ovarian cancer. We performed various statistical analyses to evaluate the effects of *CX3CL1* and *CX3CR1* genetic variants, demographic data, and clinical characteristics on the effect of carboplatin therapy. The results show that the *CX3CL1* genotypes *rs223815* (*G>C*) and *rs682082* (*G>A*) will significantly affect the clinical efficacy of carboplatin for ovarian cancer (*p* < 0.05), while the other six genotypes and all *CX3CR1* genotypes have no significant effect (*p* > 0.05). In addition, only one population factor, age, had a significant effect on the clinical efficacy of carboplatin-treated ovarian cancer (*p* < 0.05). Based on the above research results, we concluded that the clinical efficacy of carboplatin in ovarian cancer patients was significantly correlated with age and *CX3CL1* polymorphism factors; however, more in-depth effects and mechanisms need to be explored by large-scale, multicenter studies.

## 1 Introduction

Ovarian cancer is one of the most common gynecological malignancies and is mainly divided into epithelial ovarian cancer and non-epithelial ovarian cancer. According to the statistics of the World Health Organization (WHO), there are more than 239,000 new cases of ovarian cancer and 152,000 deaths worldwide each year, which directly leads to ovarian cancer ranking first in the mortality rate of malignant tumors in the female reproductive system (Gaona-Luviano et al., 2020). In China, more than 52,000 patients are diagnosed with ovarian cancer each year, which directly leads to approximately 22,500 deaths (Cao et al., 2021; He et al., 2021). Based on this, the prevention and treatment of ovarian cancer need urgent attention. Unfortunately, the special anatomical location of ovarian cancer makes the disease insidious, coupled with the lack of obvious symptoms in the initial stage, which causes 70% of patients to be diagnosed in an advanced phase, directly leading to an unsatisfactory treatment effect (Menon et al., 2018). Moreover, the current treatment of ovarian cancer is still mainly surgery supplemented by chemotherapy, biological targeted drug therapy and endocrine therapy. Although certain clinical results have been achieved, the 5-year survival rate of advanced patients is still very low, and it is very prone to recurrence and drug resistance (Morand et al., 2021). Therefore, more research is needed to screen more suitable diagnostic biomarkers, explore more valuable therapeutic targets, develop more promising therapeutic strategies, and provide new insights and directions for ovarian cancer research, clinical diagnosis and treatment.

Fractalkine (FKN, CX3CL1) is the only CX3C chemokine that can drive the development of various diseases by activating the specific receptor CX3CR1 to form the CX3CL1/CX3CR1 axis (Helmke et al., 2019; Korbecki et al., 2020; Liu et al., 2016). One study found that CX3CL1 was expressed in ovarian epithelial cancer cells and detected in malignant ascites of patients as a novel malignant cell proliferation regulator (Gaudin et al., 2011). CX3CR1 was also found to be expressed in more than 64% of metastatic ovarian cancer specimens, and it promotes ovarian cancer cell adhesion to the mesothelial monolayer and proliferation in a CX3CL1-dependent manner (Gurler et al., 2017; Kim et al., 2012). Based on this, in-depth exploration of the function and mechanism of the CX3CL1/CX3CR1 axis in ovarian cancer will provide a strong theoretical basis for the targeted therapy of ovarian cancer. Meanwhile, the CX3CL1/CX3CR1 coding region was found to have SNPs with various amino acid substitutions, such as the *T280M* SNP and *V249I* SNP, which would affect their expression, function and activity and may cause platinum resistance in ovarian cancer patients (Erreni et al., 2016; Yao et al., 2014). Therefore, it is very important to explore whether the *CX3CL1/CX3CR1* gene polymorphism is significantly related to the clinical treatment of ovarian cancer and to screen alleles related to clinical efficacy for the individualized treatment of ovarian cancer.

In this study, we collected relevant data of carboplatin-treated ovarian cancer patients, such as age and disease type, and detected the related *CX3CL1/CX3CR1* SNPs by Sequenom Mass ARRAY technology (based on the Sequenom platform) to evaluate whether they are efficacy-related alleles, determine the correlation between the above indicators and clinical efficacy, and provide a basis for the individualization of ovarian cancer.

## 2 Materials and methods

### 2.1 Patients and chemotherapy regimens

This study was carried out in strict accordance with the clinical trial management standards, and all the patients signed the informed consent form by themselves or their family members after being fully informed, which was approved by the Ethics Committee of Anhui Cancer Hospital (No. 2021-YJK-36).

**FIGURE 1:**
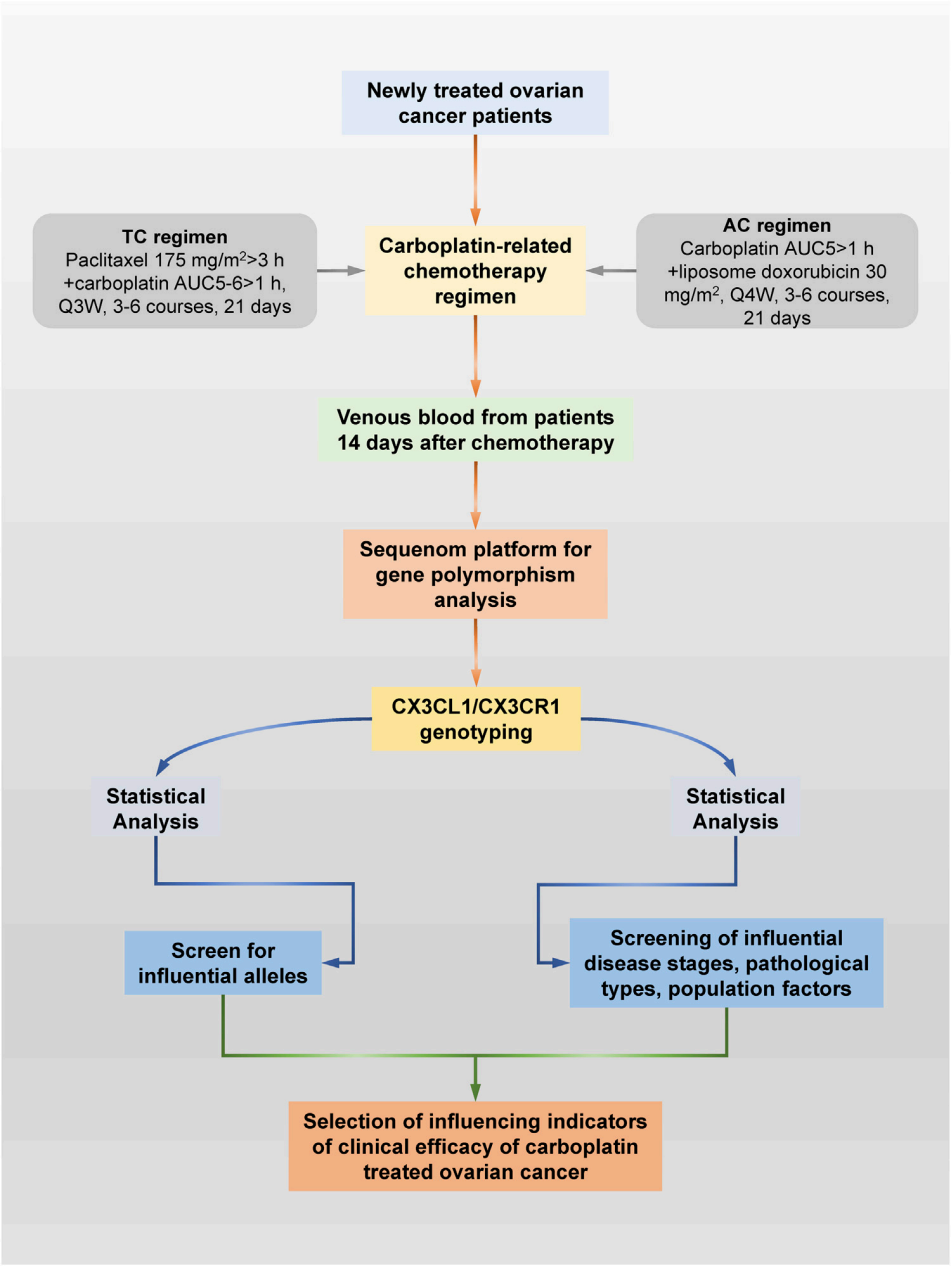
Specific research process schematic diagram. This study explored ovarian cancer patients who received carboplatin and its related drugs for the first time through pharmacogenomics technology based on Sequenom Mass ARRAY technology. Valuable ovarian cancer patients were screened according to a series of strict inclusion and exclusion criteria. Blood remaining after clinical testing was collected from all patients for genetic polymorphism testing. At the same time, the pathological type, disease stage, liver and kidney function and group indicators of all patients were obtained through the hospital information system. Finally, the significant specific *CX3CL1/CX3CR1 SNPs* and other factors that affect the chemotherapy effect of carboplatin and related drugs were screened out through statistical analysis.

A total of 384 patients with ovarian cancer from the Chinese Han population who underwent surgical treatment in the Department of Gynecologic Oncology of Anhui Cancer Hospital from January 2021 to December 2021 and were diagnosed by postoperative histopathology were selected. Patients were screened strictly according to the inclusion and exclusion criteria. Subsequently, basic demographic data, such as age and clinical information, including biochemical indicators, pathological type and disease stage, were collected and recorded for 127 eligible patients. Carboplatin chemotherapy regimens included the following: 1. Paclitaxel/carboplatin (TC) regimen: paclitaxel 175 mg/m^2^ > 3 h + carboplatin AUC5-6 > 1 h, Q3 W, three to six courses of treatment, 21 days; 2. Liposome doxorubicin/carboplatin (AC) regimen: carboplatin AUC5>1 h + liposome doxorubicin 30 mg/m^2^, Q4 W, three to six courses of treatment, 21 days of treatment. The specific research procedure is shown in Figure 1.

### 2.2 Inclusion and exclusion criteria

#### 2.2.1 Inclusion criteria

1) The patients were Chinese Han females aged 18 and above. 2) Patients diagnosed with ovarian cancer by histopathological examination. 3) The patient had not received systemic treatment for ovarian cancer before. 4) Carboplatin-based systematic and standardized chemotherapy in the Department of Gynecology and Oncology of Anhui Cancer Hospital. 5) At least one measurable lesion meeting RECIST v1.1 criteria. 6) Expected survival period ≥18 weeks 7. ECOG-PS score: 0–1 points. 8) The function of major organs is normal before chemotherapy. 9) The research patients voluntarily joined the study, signed the informed consent form, had good compliance, and cooperated with the follow-up.

#### 2.2.2 Exclusion criteria

1) Patients with recurrence and/or other malignant tumors after previous systemic treatment of ovarian cancer. 2) Patients with systemic multiple organ failure. 3) Patients with past or current central nervous system disorders. 4) Patients who participate in clinical trials of other drugs and use other clinical trial drugs for antitumor drug treatment. 5) Patients with known hypersensitivity to carboplatin. 6) Patients with a known history of psychotropic substance abuse or drug use; 7) According to the researcher’s judgment, the research patient has other factors that may lead to the termination of the study, such as other serious diseases or serious laboratory abnormalities or accompanied by other family or social factors that will affect the safety of the investigator or the collection of experimental data and samples.

### 2.3 Demographic characteristics and clinical data collection

CT scans and ovarian cancer tumor marker tests (serum CA125, serum HE4) were performed after three courses of treatment, and clinical indicators such as red blood count (RBC, 10^12/L), hemoglobin (HGB, g/l), total protein (TP, g/l), total bilirubin (TBIL, μmol/L), alanine transaminase (ALT, IU/L), aspartate transaminase (AST, IU/L), urea nitrogen (BUN, mmol/L), and creatinine (CREA, μmol/L) levels were also recorded. Meanwhile, demographic data such as the age, height, and weight of patients were recorded truthfully and accurately. Efficacy evaluation according to WHO criteria: Ⅰ. Complete remission (CR): All target lesions disappeared and lasted for 4 weeks, and serum CA125 and HE4 levels were significantly reduced; Ⅱ. Partial remission (PR): The sum of the largest tumor diameters was reduced by at least 50% and maintained over 4 weeks, serum CA125 and HE4 levels decreased; Ⅲ. Progressive disease (PD): The sum of the long diameter of the baseline lesions increased by ≥ 20% or new lesions appeared, and the serum CA125 and HE4 levels increased significantly; Ⅳ. Stabilized disease (SD): The reduction did not reach PR or increase did not reach PD, serum CA125 and HE4 levels did not increase significantly. CR and PR patients must have confirmed disease progression after 4 weeks.

### 2.4 Gene polymorphism analysis

Venous whole blood was collected from the patients on the 14th day after the first carboplatin treatment, and genomic DNA was extracted from the blood samples using the Flexi Gene DNA extraction kit (Qiagen, Germany). A Biodrop ultratrace protein accounting analyzer (BioDrop, United Kingdom) was used to accurately determine the concentration and purity of each DNA sample, and the sample DNA was concentrated by a vacuum concentrator to ensure that the final detection concentration was between 15 ng/μL and 20 ng/μL. The optical density (OD)-value (A260/A280 ratio) was between 1.8 and 2.0. All patients were genotyped for *CX3CL1* [*rs8102 (C>T), rs170361 (G>A), rs170364 (G>T), rs183419 (T>C), rs223815 (G>C), rs614230 (T>C), rs682082 (G>A), rs2239354 (G>A) and rs4151117 (G>T)*] and *CX3CR1* [*rs1050592 (G>A), rs2669841 (T>C), rs2669850 (G>C), rs2853711 (G>T), rs2853712 (T>C), rs3732378 (A>G), rs3732379 (C>T), rs4423707 (T>C), rs9826296 (A>G), rs11715522 (C>A), rs12636547 (G>C), rs13062158 (C>T) and rs17793056* (*C>T*)] using Sequenom Mass ARRAY analyzer (Sequenom, Inc., UAS). The Sequenom Mass ARRAY reaction volume was 4 μL, including 1 μL template DNA, 1 μL dNTP Mixture (TaKaRa, Japan), 0.1 μL HotstarTaq and 0.625 μL 10×PCR Buffer (Qiagen, Germany), 0.325 μL MgCl_2_ (25 mM) and 0.95 μL H_2_O. PCR amplification was performed using a high-throughput PCR machine (GeneAmp PCR System 9700, Applied Biosystems, United States) according to the instrument operation guide. The primers used are listed in Supplementary Table S1.

### 2.5 Data processing and statistical analysis

SHEsis software (http://analysis.bio-x.cn/myAnalysis.php) was used to test the allele frequency and genetic fit to analyze whether the genotype distribution conformed to Hardy-Weinberg (HW) equilibrium. Measurement data included age, weight and other data, unless otherwise stated, in the form of the mean ± S.D. Data were assessed using SPSS 26.0 (IBM Corporation, Armonk, NY, United States). For the frequency distribution of the combination of two categorical variables at each level, the chi-square analysis or Fisher’s exact test, with odds ratios (OR) and 95% confidence intervals (95% CI), was mainly used to compare the difference in rates between different groups. For the continuous variables with normal distribution, the Shapiro–Wilk test method and the single-sample Kolmogorov–Smirnov test method were used, and for non-normal distribution, the Mann–Whitney *U* test was used; the Kruskal–Wallis test was used for non-normally distributed variables data. *p < 0.05* was considered statistically significant.

## 3 Results

### 3.1 Demographic and clinical characteristics of carboplatin-treated ovarian cancer patients

After the evaluation and screening of the inclusion and exclusion criteria, a total of 127 carboplatin-treated ovarian cancer patients entered the clinical research project. The relevant demographics, biochemical indicators and clinical characteristics are shown in Table 1. Specifically, 110 ovarian cancer patients (86.61%) were treated with chemotherapy regimen 1, and 17 ovarian cancer patients (13.38%) were treated with regimen 2. There was no significant difference between age (54.5 ± 9.5 *vs.* 54.4 ± 4.7), height (159.2 ± 5.3 *vs.* 158.4 ± 6.9), weight (59.4 ± 9.5 *vs.* 60.7 ± 6.9), disease stage, disease type and treatment effect of patients within the two chemotherapy regimens, which indicated that the clinical effects of the two chemotherapy regimens were consistent, but it is clear that more doctors tend to choose chemotherapy regimen 1. Meanwhile, the clinical indicators of ovarian cancer patients treated with two chemotherapy regimens, including the values of RBC, HGB, TP, TBIL, ALT, AST, BUN, and CREA, are registered in Table 1. Statistical analysis showed that the two regimens only had significant differences in the TP index (*p* < 0.01). Specifically, the level of TP in patients with chemotherapy regimen two was significantly higher than that in patients with chemotherapy regimen 1.

**TABLE 1:** Demographic and clinical characteristics of carboplatin-treated ovarian cancer patients.

Variable	Typing and grading	Chemotherapy regimen 1	Chemotherapy regimen 2
Number of Patients, n (%)	—	110 (86.61%)	17 (13.38%)
Age (year, Mean ± SD)	—	54.5 ± 9.5	54.4 ± 4.7
Height (cm, Mean ± SD)	—	159.2 ± 5.3	158.4 ± 6.9
Weight (kg, Mean ± SD)	—	59.4 ± 9.5	60.7 ± 6.9
RBC (10^12/L, Mean ± SD)	—	3.70 ± 0.68	3.72 ± 0.58
HGB (g/l, Mean ± SD)	—	116.7 ± 13.8	114.8 ± 13.2
TP (g/l, Mean ± SD)	—	8.29 ± 6.44	9.91 ± 5.38**
TBIL (μmol/l, Mean ± SD)	—	70.98 ± 8.18	77.62 ± 8.62
ALT (IU/l, Mean ± SD)	—	18.8 ± 9.5	27.2 ± 27.6
AST (IU/l, Mean ± SD)	—	21.1 ± 6.6	26.4 ± 14.7
BUN (mmol/l, Mean ± SD)	—	5.40 ± 1.48	4.87 ± 1.06
CREA (μmol/l, Mean ± SD)	—	59.4 ± 12.5	63.4 ± 9.6
Disease type, n (%)	HGSC	98 (89.09%)	14 (82.35%)
	Other type	12 (10.91%)	3 (17.65%)
Disease stage, n (%)	I	6 (5.45%)	2 (11.76%)
	II	8 (7.27%)	3 (17.65%)
	III	84 (76.36%)	7 (41.18%)
	IV	12 (10.91%)	5 (29.41%)
Clinical efficacy, n (%)	CR	62 (56.36)	11 (64.70%)
	PR	26 (23.64)	4 (23.53%)
	“SD”	17 (15.45%)	1 (5.88%)
	PD	5 (4.54%)	1 (5.88%)

Note: Chemotherapy regimen 1 (TC, regimen): paclitaxel 175 mg/m^2^ > 3 h + carboplatin AUC5-6 > 1 h, Q3 W, three to six courses of treatment, 21 days; chemotherapy regimen 2 (AC, regimen): carboplatin AUC5>1 h + liposome doxorubicin 30 mg/m^2^.

Q4 W, three to six courses of treatment, 21 days.

Abbreviations: AC, liposome doxorubicin/carboplatin; ALT, alanine transaminase; AST, aspartate aminotransferase; AUC, area under the curve; BUN, blood urea nitrogen; CR, complete remission; CREA, creatinine; HGB, hemoglobin concentration; HGSC, high-grade serous carcinoma; PD, progressive disease; PR, partial remission; RBC, red blood count; SD, standard deviation; “SD”, stabilized disease; TBIL, total bilirubin; TC, paclitaxel/carboplatin; TP, total protein.

### 3.2 Genotypes and allele frequency of *CX3CL1* SNPs in carboplatin-treated ovarian cancer patients

After SNP detection, the distribution of genotypes and allele frequencies of *CX3CL1* [*rs8102 (C>T), rs170361 (G>A), rs170364 (G>T), rs183419 (T>C), rs223815 (G>C), rs614230 (T>C), rs682082 (G>A), rs2239354 (G>A) and rs4151117 (G>T)*] are shown in Table 2. Specifically, according to the mutation rate, the frequencies of the variant alleles of *CX3CL1* were *rs682082 (G>A)* (95.28%)*, rs4151117 (G>T)* (62.99%)*, rs8102 (C>T)* (62.60%)*, rs170364 (G>T)* (46.85%)*, rs614230 (T>C)* (41.73%)*, rs170361 (G>A)* (30.31%)*, rs183419 (T>C)* (29.13%)*, rs2239354 (G>A)* (7.09%) *and rs223815 (G>C)* (5.12%).

**TABLE 2:** Genotypes and allele frequency of *CX3CL1/CX3CR1* SNPs in carboplatin-treated ovarian cancer patients (*n* = 127).

Gene Name	SNPs	Alternative nomenclature	Genotype frequencies, n (%)			Allele frequencies (%)	
*CX3CL1*	rs170361	G>A	GG	GA	AA	G (69.68)	A (30.31)
			61 (48.03)	55 (43.31)	11 (8.66)		
	rs170364	G>T	GG	GT	TT	G (53.15)	T (46.85)
			62 (48.82)	11 (8.66)	54 (42.52)		
	rs183419	T>C	TT	TC	CC	T (70.87)	C (29.13)
			64 (50.39)	52 (40.94)	11 (8.66)		
	rs223815	G>C	GG	GC	CC	G (94.88)	C (5.12)
			115 (90.55)	11 (8.66)	1 (0.79)		
	rs2239354	G>A	GG	GA	AA	G (92.91)	A (7.09)
			109 (85.83)	18 (14.17)	0 (0.00)		
	rs4151117	G>T	GG	GT	TT	G (30.01)	T (62.99)
			17 (13.38)	60 (47.24)	50 (39.37)		
	rs614230	T>C	TT	TC	CC	T (58.27)	C (41.73)
			43 (33.86)	62 (48.82)	22 (17.32)		
	rs682082	G>A	GG	GA	AA	G (4.72)	A (95.28)
			1 (0.79)	10 (7.87)	116 (91.34)		
	rs8102	C>T	CC	CT	TT	C (37.40)	T (62.60)
			18 (14.17)	59 (46.47)	50 (39.37)		
*CX3CR1*	rs1050592	G>A	AA	AG	GG	A (97.24)	G (2.76)
			120 (94.49)	7 (5.51)	0 (0.00)		
	rs11715522	C>A	CC	CA	AA	C (63.78)	A (36.22)
			48 (37.80)	66 (51.97)	13 (10.24)		
	rs12636547	G>C	GG	GC	CC	G (94.49)	C (5.51)
			114 (89.76)	12 (9.45)	1 (0.79)		
	rs13062158	C>T	CC	CT	TT	C (63.38)	T (36.61)
			48 (37.80)	65 (51.18)	14 (11.02)		
	rs17793056	C>T	CC	CT	TT	C (33.86)	T (65.35)
			13 (10.24)	60 (47.24)	53 (41.73)		
	rs2669841	T>C	TT	TC	CC	T (13.78)	C (86.22)
			3 (2.36)	29 (22.83)	95 (74.80)		
	rs2669850	G>C	GG	GC	CC	G (74.02)	C (25.98)
			69 (54.33)	50 (39.37)	8 (6.30)		
	rs2853711	G>T	GG	GT	TT	G (86.22)	T (13.78)
			95 (74.80)	29 (22.83)	3 (2.36)		
	rs2853712	T>C	TT	TC	CC	T (77.16)	C (22.83)
			74 (58.27)	48 (37.80)	5 (3.94)		
	rs3732378	A>G	AA	AG	GG	A (2.76)	G (97.24)
			0 (0.00)	7 (5.51)	120 (94.49)		
	rs3732379	C>T	CC	CT	TT	C (97.24)	T (2.76)
			120 (94.49)	7 (5.51)	0 (0.00)		
	rs4423707	T>C	TT	TC	CC	T (5.12)	C (94.88)
			1 (0.79)	11 (8.66)	115 (90.55)		
	rs9826296	A>G	AA	AG	GG	A (22.05)	G (77.95)
			4 (3.15)	48 (37.80)	75 (59.06)		
	rs9868689	NA	CC	NA	NA	C (100)	NA
			127 (100)				

### 3.3 Genotypes and allele frequency of *CX3CR1* SNPs in carboplatin-treated ovarian cancer patients

The distribution of genotypes and allele frequencies of *CX3CR1* [*rs1050592 (G>A), rs11715522 (C>A), rs12636547 (G>C), rs13062158 (C>T), rs17793056 (C>T), rs2669841 (T>C), rs2669850 (G>C), rs2853711 (G>T), rs2853712 (T>C), rs3732378 (A>G), rs3732379 (C>T), rs4423707 (T>C)* and *rs9826296 (A>G)*] are shown in Table 2. Specifically, according to the mutation rate, the frequencies of the variant alleles of *CX3CR1* were *rs3732378 (A>G) (97.24%), rs4423707 (T>C)* (94.88%)*, rs2669841 (T>C)* (86.22%)*, rs9826296 (A>G)* (77.95%)*, rs17793056 (C>T)* (65.35%)*, rs13062158 (C>T)* (36.61%)*, rs11715522 (C>A)* (36.22%)*, rs2669850 (G>C)* (25.98%)*, rs2853712 (T>C)* (22.83%)*, rs2853711 (G>T)* (13.78%)*, rs12636547 (G>C)* (5.51%)*, rs1050592 (G>A)* (2.76%) and *rs3732379 (C>T)* (2.76%). Meanwhile, no allelic changes were detected at the rs9868689 SNP locus of the *CX3CR1* genotype.

### 3.4 Influences of *CX3CL1/CX3CR1* SNPs on the clinical efficacy of carboplatin-treated ovarian cancer patients

We examined a total of 22 genotypes of *CX3CL1* and *CX3CR1* to explore the association between different genotypes and the clinical efficacy of carboplatin treatment in ovarian cancer patients. The effects of *CX3CL1* and *CX3CR1* SNPs on the clinical efficacy of carboplatin in the treatment of ovarian cancer are shown in Table 3. The results showed that the CX3CL1 genotypes *rs223815* (*χ* ^*2*^ = 6.592, *p* = 0.037) and *rs682082* (*χ* ^*2*^ = 7.025, *p* = 0.030) were closely related to the clinical efficacy of carboplatin treatment for ovarian cancer. Specifically, the *G>C* mutation at *rs223815* and the *G>A* mutation at *rs682082* will significantly improve the therapeutic effect. Meanwhile, a subset of *CX3CL1* SNPs [*rs170361 (G>A)*, *rs170361 (G>A), rs183419 (T>C)*, *rs8102 (C>T), rs614230 (T>C), rs2239354 (G>A)* and *rs4151117 (G>T)*] and all *CX3CR1* SNPs showed no significant association with carboplatin therapy-related clinical efficacy in ovarian cancer patients.

**TABLE 3:** Association of *CX3CL1* and *CX3CR1* SNPs with the clinical efficacy of carboplatin treatment in ovarian cancer patients.

Gene	SNP	Genotype [CR/PR]/[PD/SD]	χ^2^	*p*-Value
*CX3CL1*	rs170361	GG [46]/[15]	4.083	0.130
		GA [46]/[9]		
		AA [11]/[0]		
	rs170364	GG [47]/[15]	4.277	0.118
		GT [45]/[8]		
		TT [11]/[0]		
	rs183419	TT [48]/[16]	4.537	0.103
		TC [44]/[8]		
		CC [11]/[0]		
	rs223815	GG [97]/[20]	6.592	0.037
		GC [5]/[5]		
		CC [1]/[0]		
	rs2239354	GG [88]/[21]	0.068	0.794
		GA [15]/[3]		
		AA [0]/[0]		
	rs4151117	GG [16]/[1]	3.719	0.156
		GT [50]/[10]		
		TT [37]/[13]		
	rs614230	TT [32]/[11]	2.037	0.361
		TC [53]/[9]		
		CC [18]/[4]		
	rs682082	GG [1]/[0]	7.025	0.030
		GA [5]/[5]		
		AA [97]/[19]		
	rs8102	CC [17]/[1]	3.882	0.144
		CT [49]/[10]		
		TT [37]/[13]		
*CX3CR1*	rs1050592	AA [98]/[22]	0.452	0.501
		AG [5]/[2]		
		GG [0]/[0]		
	rs11715522	CC [40]/[8]	0.491	0.782
		CA [11]/[2]		
		AA [52]/[14]		
	rs12636547	GG [92]/[22]	0.284	0.868
		GC [10]/[2]		
		CC [1]/[0]		
	rs13062158	CC [40]/[7]	0.973	0.615
		CT [51]/[14]		
		TT [12]/[2]		
	rs17793056	CC [11]/[3]	0.859	0.651
		CT [47]/[13]		
		TT [45]/[8]		
	rs2669841	TT [3]/[0]	0.760	0.684
		TC [23]/[6]		
		CC [77]/[18]		
	rs2669850	GG [57]/[11]	2.185	0.335
		GC [41]/[9]		
		CC [5]/[3]		
	rs2853711	GG [77]/[18]	0.760	0.518
		GT [23]/[6]		
		TT [3]/[0]		
	rs2853712	TT [62]/[12]	2.678	0.262
		TC [36]/[12]		
		CC [5]/[0]		
	rs3732378	AA [0]/[0]	0.452	0.501
		AG [5]/[2]		
		GG [98]/[22]		
	rs3732379	CC [98]/[22]	0.452	0.501
		CT [5]/[2]		
		TT [0]/[0]		
	rs4423707	TT [1]/[0]	0.241	0.887
		TC [9]/[2]		
		CC [93]/[22]		
	rs9826296	AA [3]/[1]	0.172	0.918
		AG [39]/[9]		
		GG [62]/[13]		

Abbreviations: CR, complete remission; PD, progressive disease; PR, partial remission; “SD”, stabilized disease.

### 3.5 Influence of population factors on the clinical efficacy of carboplatin in patients with ovarian cancer

After examining the clinical efficacy of all SNPs of *CX3CL1/CX3CR1* on carboplatin-treated ovarian cancer patients, we comprehensively evaluated the effect of demographic factors, clinical indicators and SNPs on carboplatin-treated ovarian cancer patients by the Mann–Whitney *U* test and χ^2^ test, and the results are shown in Table 4. In addition to the abovementioned SNPs that may significantly improve clinical efficacy, the analysis also showed that only the age factor (*Z* = −2.186, *p* = 0.029) affected the chemotherapy effect of carboplatin, which proved to be a negative correlation with the therapeutic effect.

**TABLE 4:** Influence of population factors on the clinical therapeutic effect of carboplatin in patients with ovarian cancer.

Variable	Typing/grading	CR/PR	PD/SD	*Z*/χ^2^	*p*-Value
Number of Patients, n (%)	—	103 (81.10%)	24 (18.90%)	—	—
Age (year, mean ± SD)	—	55.583 ± 8.007	49.917 ± 11.587	−2.186	0.029
Height (cm, mean ± SD)	—	159.039 ± 5.928	159.208 ± 3.695	−0.533	0.594
Weight (kg, mean ± SD)	—	60.049 ± 9.421	57.417 ± 7.912	−0.909	0.363
RBC (10^12/L, mean ± SD)	—	3.638 ± 0.599	3.990 ± 0.868	−1.872	0.061
HGB (g/L, mean ± SD)	—	116.252 ± 13.672	117.125 ± 13.973	−0.071	0.944
TP (g/L, mean ± SD)	—	71.416 ± 8.321	73.808 ± 9.249	−1.432	0.152
TBIL (μmol/L, mean ± SD)	—	8.877 ± 6.835	6.938 ± 2.869	−0.659	0.510
ALT (IU/L, mean ± SD)	—	20.660 ± 14.589	16.875 ± 6.701	−1.160	0.246
AST (IU/L, mean ± SD)	—	22.029 ± 8.102	20.917 ± 9.079	−1.130	0.259
BUN (mmol/L, mean ± D)	—	5.409 ± 1.492	4.967 ± 1.124	−1.534	0.125
CREA (μmol/L, mean ± SD)	—	60.198 ± 12.185	58.667 ± 12.218	−0.419	0.675
Disease type, n (%)	HGSC	92	23	0.965	0.326
	Other type	11	1		
Disease stage, n (%)	I	3	0	4.157	0.245
	II	3	0		
	III	76	15		
	IV	21	9		
Chemotherapy regimen, n (%)	1	88	22	0.652	0.420
	2	15	2		

Note: Chemotherapy regimen 1 (TC, regimen): paclitaxel 175 mg/m^2^ > 3 h + carboplatin AUC5-6 > 1 h, Q3 W, three to six courses of treatment, 21 days; chemotherapy regimen 2 (AC, regimen): carboplatin AUC5>1 h + liposome doxorubicin 30 mg/m^2^.

Q4 W, three to six courses of treatment, 21 days.

Abbreviations: AC, liposome doxorubicin/carboplatin; ALT, alanine transaminase; AST, aspartate aminotransferase; AUC, area under the curve; BUN, blood urea nitrogen; CR, complete remission; CREA, creatinine; HGB, hemoglobin concentration; HGSC, high-grade serous carcinoma; PD, progressive disease; PR, partial remission; RBC, red blood count; SD, standard deviation; “SD”, stabilized disease; TBIL, total bilirubin; TC, paclitaxel/carboplatin; TP, total protein.

## 4 Discussion

As one of the most common malignant tumors in the female reproductive system, the occurrence and development of ovarian cancer seriously threaten women’s health and bring heavy economic and social burdens. Therefore, the prevention and treatment of ovarian cancer is particularly important. In addition to surgical resection, current clinical treatments for ovarian cancer include chemotherapy, radiotherapy, and immunotherapy (An et al., 2021). A study of chemotherapy for ovarian cancer found that although paclitaxel combined with platinum is the most preferred regimen, many patients still die due to recurrence or chemical toxicity, while carboplatin combined with liposomal doxorubicin has gradually been clinically recognized due to its significant therapeutic effect (An et al., 2021; Lheureux et al., 2019). However, to date, platinum-based paclitaxel drugs and platinum-based liposomal doxorubicin are still the first-line chemotherapy regimens for clinical ovarian cancer. In this study, we selected ovarian cancer patients who were treated in our hospital from January to December 2020 to conduct relevant clinical trials to explore the clinical efficacy of *CX3CL1/CX3CR1* gene polymorphisms in carboplatin-treated patients. After strict screening with preestablished inclusion and exclusion criteria, 127 ovarian cancer patients who received carboplatin and related treatments entered the study. We found that 110 patients were treated with chemotherapy regimen 1, that is, TC regimen accounted for 86.6%, and 17 patients were treated with chemotherapy regimen 2, that is, AC regimen accounted for 13.4%, and there was no significant difference in the two treatment regimens in terms of hematology and liver and kidney toxicity. The above results suggest that when choosing a chemotherapy regimen for ovarian cancer patients, two regimens can be flexibly selected according to the patient’s own conditions, disease development status, drug compatibility and other factors to maximize the implementation of individualized treatment. Meanwhile, we should also understand that whether it is chemotherapy regimen one or 2, the most serious toxic effect is bone marrow transplantation. Therefore, in follow-up studies, more attention will be focused on toxic effects, such as bone marrow transplantation.

Pharmacogenomics is an important means to implement individualized and precise treatment of diseases (Piyawajanusorn et al., 2021). It mainly detects the changes in genotype and phenotype in patients’ tissues or body fluids through a variety of methods and then uses the relevant gene theory to comprehensively explore the differences and mechanisms of genomic changes in response to drugs, which will provide an effective science for avoiding irrational drug use and ineffective treatment (Weinshilboum and Wang, 2017). Among them, genome-wide association studies (GWAS) have shown that genetic variants are associated with ovarian cancer, and single nucleotide polymorphisms (SNPs), also known as gene polymorphisms, are particularly important (Lawrenson et al., 2019; Zhou et al., 2020). Genetic testing of 1,342 ovarian cancer patients revealed that 176 patients had *BRCA* gene mutations, including 107 *BRCA1* mutations and 67 *BRCA2* mutations, and two mutations in both genes, accounting for 13.3% of the comprehensive mutation frequency (Kristeleit et al., 2022; Zhang et al., 2011). Moreover, some genetic variants are shared, but there are also certain population specificities, such as *WNT4*, *ESR2*, and *PSEN1* gene polymorphisms, which have been reported to be involved in ovarian cancer in Chinese individuals (Feng et al., 2019; Xu et al., 2018; Zhang et al., 2018). A functional study found that the TT genotype of the *ERCC1* gene seems to be beneficial to the better clinical effect of platinum-based chemotherapy (Steffensen et al., 2008). Meanwhile, the exon 21 *MDR-1* polymorphism G2677T/A was confirmed to be associated with the paclitaxel response in ovarian cancer, including P-glycoprotein function and paclitaxel resistance, which provides useful information for individualized therapy of ovarian cancer (Green et al., 2006). Therefore, in-depth research and attempts to clarify the relationship between gene polymorphisms and ovarian cancer and explore the relevant mechanism will provide important ideas, clues and foundations for the establishment of ovarian cancer biomarkers, clinical diagnosis and targeted therapy. At present, studies have reported that the CX3CL1/CX3CR1 biological axis can affect the drug resistance of ovarian cancer cells by mediating changes in the levels of inflammatory factors and cytokines, thereby maintaining the sensitivity of tumor cells to platinum drugs (Liu et al., 2021; Miao et al., 2020). Meanwhile, it has also been found that CX3CL1 exerts the aforementioned effects, which may be closely related to the fact that its binding to CX3CR1 induces the activation of heterotrimeric G proteins associated with this receptor and activates the mitogen-activated protein kinase (MAPK) and protein kinase B (Akt) signaling pathways (Rivas-Fuentes et al., 2021). As research continues to advance, the role of the CX3CL1/CX3CR1 biological axis in tumors is gradually being explored, but the roles of their gene polymorphisms in ovarian cancer are relatively unknown and require more exploration. Therefore, in this study, we used Sequenom Mass ARRAY technology to perform high-throughput analysis of *CX3CL1* and *CX3CR1* gene polymorphisms, aiming to explore the effect of *CX3CL1* and *CX3CR1* gene polymorphisms on the clinical efficacy of carboplatin in the treatment of ovarian cancer. Through analysis, we found that a total of 22 single nucleotide polymorphisms were detected in the DNA of carboplatin-treated ovarian cancer patients, including nine *CX3CL1* SNPs [*rs8102 (C>T), rs170361 (G>A), rs170364 (G > T), rs183419 (T>C), rs223815 (G>C), rs614230 (T>C), rs682082 (G>A), rs2239354 (G>A)* and *rs4151117 (G>T)*] and 13 *CX3CR1* SNPs [*rs1050592 (G>A), rs11715522 (C>A), rs12636547 (G>C), rs13062158 (C>T), rs17793056 (C>T), rs2669841 (T>C), rs2669850 (G>C), rs2853711 (G>T), rs2853712 (T>C), rs3732378 (A>G), rs3732379 (C>T), rs4423707 (T>C)* and *rs9826296 (A>G)*], which suggests that the *CX3CL1/CX3CR1* gene polymorphism is likely to be involved in affecting the clinical efficacy of carboplatin in the treatment of ovarian cancer, such as carboplatin resistance, *etc.* Meanwhile, statistical results showed that *CX3CL1* genotypes rs223815 (*χ* ^*2*^ = 6.592, *p* = 0.037) and rs682082 (*χ* ^*2*^ = 7.025, *p* = 0.030) are closely related to the clinical efficacy of carboplatin-treated ovarian cancer, which suggests that when we choose to use carboplatin for ovarian cancer chemotherapy, the above three SNP genotypes need to be detected to provide clinical reference. We then performed statistical analysis on all influencing factors and found that, except for three *CX3CL1* SNPs, only the age factor (*Z* = −2.186, *p* = 0.029) affected the chemotherapy effect of carboplatin, which proved to be a negative correlation with the therapeutic effect. The above results suggest that we should also fully consider the age factor while paying attention to gene polymorphisms or SNPs when using carboplatin for ovarian cancer.

In this study, we also realized that there are still some limitations and inadequacies, which mainly include the following points: 1) Since there may be great differences in genetic polymorphisms among different ethnic groups, the conclusions obtained in this study are only applicable to Han patients receiving carboplatin for ovarian cancer. 2) This study is still only a small-scale study, which may not be enough to fully explain the effect of *CX3CL1/CX3CR1* gene polymorphisms on the clinical efficacy of carboplatin for ovarian cancer patients. Therefore, more samples or multicenter samples are needed to verify the in-depth connection. 3) The evaluation of toxicity and side effects in this study is not thorough. In a follow-up study, we will add more toxicity indicators of chemotherapy drugs to comprehensively evaluate the effect of *CX3CL1/CX3CR1* gene polymorphisms on the clinical efficacy of carboplatin in patients with ovarian cancer.

## 5 Conclusion

Collectively, we demonstrated that the clinical efficacy of carboplatin in ovarian cancer patients was associated with polymorphisms of *CX3CL1* [*rs223815* (*G>C*) and *rs682082* (*G>A*)]. In addition, the influence of age on clinical efficacy cannot be ignored. The mechanism by which *CX3CL1* gene polymorphisms affect the clinical efficacy of carboplatin in patients with ovarian cancer needs to be explored by large-scale, multicenter studies.

## Data Availability

The original contributions presented in the study are included in the article/Supplementary Material, further inquiries can be directed to the corresponding author.
